# Lipidomics Reveals Differences in Lipid Composition Between Lipid Droplets and Milk Fat Globules in Dairy Goat Mammary Tissue

**DOI:** 10.3390/ani15223303

**Published:** 2025-11-15

**Authors:** Kuixian Wu, Jingna Yang, Yu Yang, Haohan Wang, Yuxin Fan, Yanbin Yang, Yang Liu, Liqiang Han

**Affiliations:** 1 Key Laboratory of Animal Biochemistry and Nutrition, Ministry of Agriculture, College of Veterinary Medicine, Henan Agricultural University, Zhengzhou 450046, China; 17739677771@163.com (K.W.); yangjingna2025@126.com (J.Y.); y5y5zi@163.com (Y.Y.);; 2 Key Laboratory of Veterinary Biotechnology of Henan Province, College of Veterinary Medicine, Henan Agricultural University, No. 15 Pingandadao Road, Zhengzhou 450046, China

**Keywords:** milk fat globules, lipid droplets, lipidomic, dairy goat

## Abstract

Milk fat globules (MFGs) are derived from lipid droplets (LDs) in the mammary epithelial cells of ruminants and released into milk. This study aimed to detect the differences in LDs and MFGs to reveal the mechanism by which LDs transform into MFGs in the mammary cells of dairy goats. The mammary gland tissue and milk of lactating dairy goats were collected and analyzed using lipidomics. The results showed that a total of 1167 and 675 lipid molecules were detected in the LDs and MFGs, respectively. The top four lipids in terms of their contents in the LDs were triglycerides (TGs), phosphatidylethanolamine (PE), phosphatidylcholine (PC), and diglycerol (DG), whereas the top four in the MFGs were TG, PE, PC, and phosphatidylinositol (PI). Furthermore, compared to the LDs, the contents of TG and DG in the MFGs were significantly increased (*p* < 0.05), while PC content was significantly decreased (*p* < 0.05). We also found that diglycerol (16:0:18), diglycerol (O-19:0:16), triglycerides (14:0:0_16:0), and phosphatidylethanolamine (P-15:0:20:3) were the key characteristic lipids in the transformation of LDs into MFGs. This study reveals the unique lipid profiles of LDs and MFGs and provides a theoretical basis for understanding the mechanism behind low-fat milk syndrome.

## Simple Summary

Milk fat globules (MFGs) are derived from intracellular lipid droplets (LDs) in the mammary epithelial cells of ruminants. This process involves the progressive maturation, apical localization, and subsequent envelopment of lipid droplets by the plasma membrane prior to their secretion into milk. This study investigated the compositional differences between LDs and MFGs, with the goal of elucidating the mechanism underlying the transformation of the former into the latter in the mammary cells of dairy goats. The results revealed that the contents of triglycerides and diglycerides in MFGs were significantly increased, while the contents of phosphatidylcholine were significantly decreased, compared with those in LDs. Additionally, we identified diglycerides (16:0_18:0), diglycerides (O-19:0_16:0), triglycerides (14:0_16:0_16:0), and phosphatidylethanolamine (P-15:0_20:3) as key characteristic lipids involved in the transformation of LDs into MFGs. Our research has uncovered the unique lipid profiles of LDs and MFGs, which may provide a theoretical foundation for understanding the mechanism underlying low-fat milk syndrome.

## 1. Introduction

Among mammals, milk is the main source of nutrition for young animals, as it contains considerable quantities of protein and lipids. It is also an indispensable source of nutrients for humans [1]. Milk fat, also known as milk fat globules (MFGs), is synthesized from lipid droplets, serving as raw materials, in mammary epithelial cells. It comprises 3% to 5% of the contents of milk on average, with the energy it provides constituting about 40% of milk’s total calories [2,3,4]. A previous study reported that milk fat is mainly composed of triglycerides (TGs) and also has a 1% content of phospholipids and small quantities of sterols, non-esterified fatty acids, and lipid-soluble vitamins, critical to the growth and development of young animals [5]. Low-fat milk syndrome refers to a marked reduction in milk fat percentage or yield in lactating dairy cows, with total milk production remaining relatively unchanged. Importantly, the types and contents of lipids in milk are closely related to its quality [6]. Therefore, studying the lipids in LDs and MFGs is of great significance for revealing the mechanism underlying low-MFG syndrome and improving milk quality.

In mammary cells, LDs are spheroid organelles formed by a membrane wrapped in a phospholipid monolayer, and they are synthesized [7] and secreted by the endoplasmic reticulum within the cell. Various types of lipids exist and are bound on the membrane’s surface [8]. Additionally, LDs are key organelles for lipid storage in cells, with the phospholipids composing their single-layer membrane structure primarily including phosphatidylcholine (PC), Phosphatidylethanolamine (PE), phosphatidylinositol (PI), and Lysophosphatidyl ethanolamine (LPE) [9,10]. It is generally acknowledged that small LDs become larger through fusion [11,12] and exert their biological functions through the combination of various proteins on their membrane with other substances [13]. After the formation of LDs by triacylglycerol and their maturation in cells, they are released into the milk through budding to form MFGs [14]. According to previous studies, MFGs are spherical structures formed by three layers of membranes, with phospholipids surrounding the triacylglycerol core [15,16]. MFGs represent an important nutrient for the early growth and cognitive development of infants [17]. Research shows that MFGs enhance immune function and resistance to infection in young animals [18], also supporting healthy growth and improved body composition [19].

MFGs are synthesized using LDs as the raw material. It is known that MFGs play a significant role in reducing infection risk in young animals and support the development of the intestinal tract and its barrier function [20]. However, the differences in lipids between LDs and MFGs in dairy goats remain unclear. This study therefore aimed to characterize these differences, thereby providing a theoretical foundation for developing MFG-based nutritional interventions.

## 2. Materials and Methods

### 2.1. Animal Ethics Statement

This study was carried out according to the Chinese guidelines for animal welfare and approved by the Animal Care and Use Committee of the Henan Agricultural University.

### 2.2. Experimental Design and Management

The experiment was carried out at the Xuchang practice base of Henan Agricultural University. A total of 12 dairy goats in good health were selected from a herd of 200 Saanen dairy goats and then numbered and marked. Milk samples were collected from the goats in the middle lactation stage (160 ± 5 d). The goats were fed at 6:00 a.m. and 16:00 p.m. and raised in a ventilated pen, with each goat occupying a single stall. A small-piston pneumatic milking machine was used to collect milk from the dairy goats at 5:00 a.m. every day. Milk samples were collected on the 160th day of lactation and divided into two parts: one used for immediate measurement of milk fat globule size with a Malvern 3000 particle size analyzer, and the other stored at −40 °C for subsequent lipid analysis. On the 28th day, all experimental goats were slaughtered and their mammary tissue was collected, rinsed with normal saline, and frozen in liquid nitrogen for storage.

### 2.3. Oil Red O Staining

LDs were stained with Oil Red O as previously described [21]. In brief, mammary tissue sections were washed with PBS and fixed in 10% formalin for 30 min. After being washed, the sections were stained for 1 h with 0.5% Oil Red O in 60% isopropanol. Following another wash, the sections were counterstained with hematoxylin for 5 min. They were then mounted and observed under an optical microscope (Olympus, Tokyo, Japan). From each image, 60 LDs were randomly selected, and their diameters were measured.

### 2.4. Staining of Breast Tissue with Nile Red

A Nile red working solution (Buffer B = 1:9, *v*/*v*) was prepared at a concentration of 100 μg/mL. A 10 μL aliquot of lipid droplets was transferred to a microcentrifuge tube, mixed with 2.5 μL of the Nile red working solution, and incubated for 10 min. Subsequently, a 2.5 μL aliquot of the mixture was placed on a glass slide and covered with a coverslip. The samples were observed under an inverted fluorescence microscope, and images were recorded for analysis [22].

### 2.5. Lipid Droplet Extraction

Lipid droplets (LDs) were extracted from breast tissue according to a differential centrifugation protocol. Briefly, approximately 5 g of breast tissue was removed from liquid nitrogen, weighed, and thawed on ice. Buffer A was then added, and the tissue was homogenized on ice using a mortar and pestle for approximately 5 min. The homogenate was centrifuged at 2500× *g* for 10 min at 4 °C. The resulting supernatant was filtered through a 100 μm cell strainer to obtain 10 mL of filtrate. A sucrose solution and Buffer B were added to this filtrate, followed by centrifugation at 4000× *g*. The upper, light-colored layer was collected in a 1.5 mL centrifuge tube and labeled LD1.

The remaining material was subjected to a series of sequential centrifugations at 4 °C: 7000× *g* for 30 min, 10,000× *g* for 30 min, 50,000× *g* for 60 min, and finally 100,000× *g* for 60 min. The pellets obtained through each of these steps were collected in separate 1.5 mL tubes and labeled LD2, LD3, LD4, and LD5, respectively. Subsequently, 200 μL of Buffer B was added to each of the tubes containing LD1 through LD5, and the samples were centrifuged at 4 °C to isolate the purified LDs. Finally, the LD pellets were washed 2–3 times with Buffer B and stored at −80 °C.

### 2.6. Lipidomics Analysis

Each mammary tissue sample was extracted and weighed. Then, 1 mL of an extract solution with an internal lipid standard was added, and the sample was mixed, homogenized with 200 μL of deionized water, and centrifuged at 12,000 r/min for 10 min at 4 °C. Subsequently, 500 μL of supernatant was placed in the tubes and concentrated until completely dry; it was then homogenized with 200 μL of a lipid solution (acetonitrile/isopropyl alcohol = 1:1, *v*/*v*), centrifuged at 4 °C, and collected for LC-MS/MS analysis [23]. Mass spectrometry was conducted under the following conditions: Electrospray ion source (Electrospray Ionization, ESI) temperature—500 °C; positive-ion-mode mass spectrometry voltage—5500 V; anion-mode mass spectrometry voltage—4500 V; and ion sources—gas 1 (GS1), 45 psi, gas 2 (GS2), 55 psi, and Curtain Gas (CUR), 35 psi. Lipid quantification was performed using the multi-reaction detection mode in triple-quadrupole mass spectrometry, in which each ion pair is scanned according to the optimized de-clustering voltage and collision energy. Based on the self-built metware database, qualitative analysis was conducted according to the retention time of detected substances. The mass spectrometry data from samples were converted into a final lipidomics dataset through data processing. This dataset can be used for both quantitative and qualitative analysis as well as data interpretation. LC–MS analysis was performed using Analyst 1.6.3, and the resulting data were normalized and converted into a 2-dimensional data matrix in Excel 2016 (Microsoft Corp., Redmond, WA, USA).

### 2.7. Statistical Analysis

The β diversity was used to investigate structural variation in the lipids through principal component analysis (PCoA) and orthogonal partial least squares discriminant analysis (OPLS-DA) based on unweighted UniFrac distance with constrained ordination. Supervised learning was performed using R software (version 4.0.2) to reveal the lipid variation among samples. The Spearman correlation between the lipid composition of LDs and MFGs was evaluated using MSTOOLS 5.0 software. A *p*-value < 0.05 was considered statistically significant for all comparisons.

## 3. Results

### 3.1. LDs in Dairy Goat Mammary Tissue

**Figure 1:**
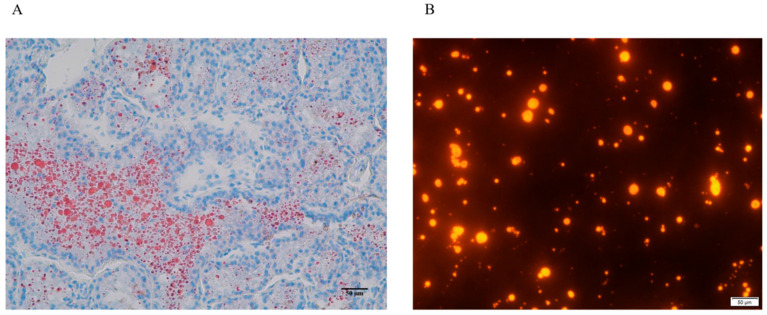
Mammary tissue sections and lipid droplet staining. (**A**) Red indicates lipid droplets stained by Oil Red O, while blue represents the nuclei; (**B**) Nile red staining of lipid droplets separated through gradient centrifugation.

As shown in Figure 1A, Oil Red O staining revealed that the mammary tissue of dairy goats contained large lipid droplets in the middle lactation period, while Nile red staining demonstrated that lipid droplets were separated from mammary epithelial cells through gradient centrifugation (Figure 1B).

### 3.2. Comparative Analysis of Lipidomics Between LDs and MFGs

Lipidomics was used to detect differences between LDs and MFGs. The results showed that a total of 1078 lipid molecules were detected in LDs, while 675 were found in MFGs, representing 45 subclasses. The LDs contain 531 types of glycerophospholipids (GPs), 360 types of glycerides (GLs), 172 types of sphingolipids (SPs), 91 types of fatty acyls (FAs), 11 types of sterol lipids (STs) and 2 types of isopentenol lipids (PR). Meanwhile, the MFGs contain 299 GPs, 271 GLs, 57 SPs, 42 FAs, and 6 STs. The top ten lipid subclasses are shown in Table 1, among which the four with the highest contents are triglycerides (TGs), phosphatidylethanolamine (PE), phosphatidylcholine (PC), and diglycerol (DG).

**Figure 2:**
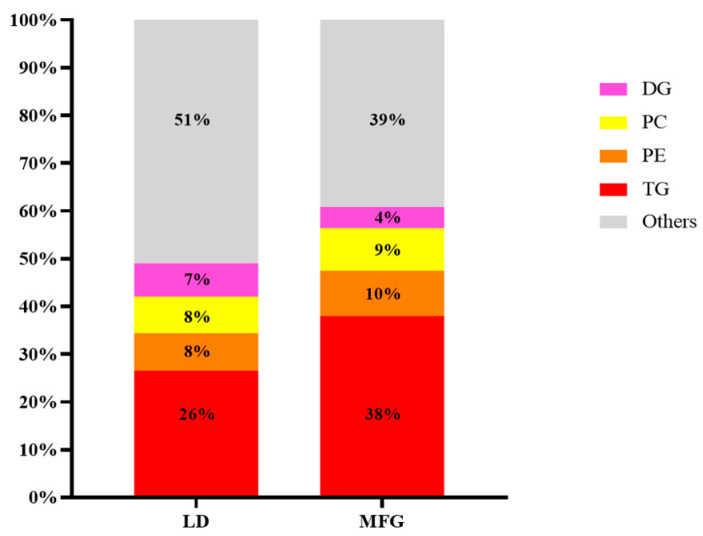
Number of lipid components in lipid droplets and milk fat globules. Percentage of each lipid species in total lipids; “Others” is the remaining lipid species after removal of TG, PE, PC, and DG.

The proportions of these four lipid subclasses were calculated and are shown in Figure 2. In LDs, the proportions were 26%, 8%, 8%, and 7%, respectively, while those in MFGs were 38%, 10%, 9%, and 4%. By comparison, in the transformation of lipid droplets into fat globules and their secretion, although the number of TG, PC, and PE types in MFGs was less than in LDs, the relative proportion these subclasses in MFGs is higher than in LDs. Meanwhile, not only the number of types but also the proportion of DG was reduced in MFGs compared to in LDs.

### 3.3. Diversity of LDs in Mammary Tissue with MFGs

**Figure 3:**
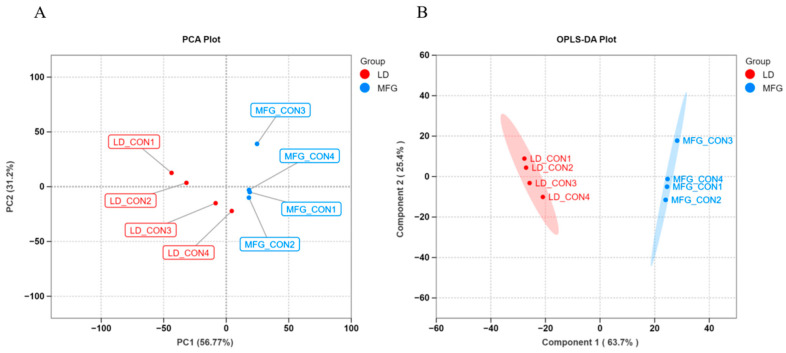
PCA and OPLS-DA of LD and MFG. (**A**) PCA was compared between the LD and MFG groups; (**B**) OPLS-DA was compared between the LD and MFG groups.

As shown in Figure 3, PCoA plots indicated a clear separation between LDs and MFGs. We thus performed an OPLS-DA, with the results showing that the two groups had clearly separated and clustered into distinct groups, which indicated that the lipids in LDs and MFGs have different relationships.

### 3.4. The Quantity of and Differences Between Each Lipid Subclass

**Figure 4:**
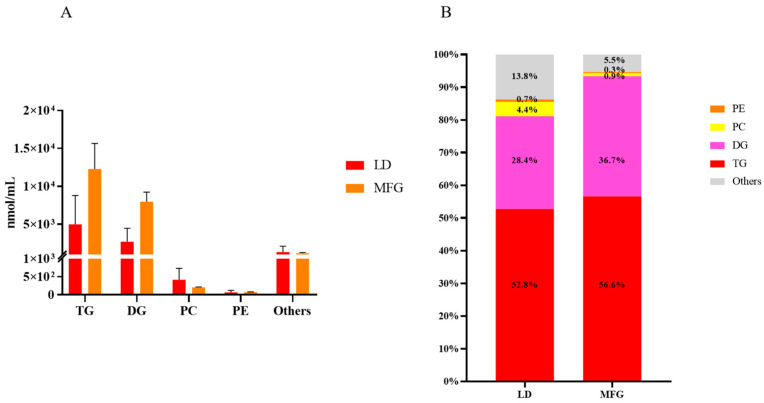
Content of each lipid subclass in lipid droplets and milk fat globules. (**A**) Contents of all lipids and each subclass; (**B**) the percentage of each lipid content in the total lipid. “Others” is the remaining lipid species after removal of TG, PE, PC, and DG.

As shown in Figure 4, compared with LDs, the total contents of TGs and DGs in MFGs increased significantly, with the former increasing from 4983.64 nmol/mL to 12,270.10 nmol/mL and the latter increasing from 2679.11 nmol/mL to 7963.28 nmol/mL. The PC content decreased from 416.25 nmol/mL to 205.75 nmol/mL, while the PE contents in LDs and MFGs were 67.69 nmol/mL and 70.96 nmol/mL, respectively. As shown in Figure 4B, compared with LDs, the TG content in MFGs increased from 52.8% to 56.6%, while the DG content increased from 28.4% to 36.7%. In addition, the percentages of PC and PE both decreased, from 4.4% to 0.9% for the former and from 0.7% to 0.3% for the latter.

### 3.5. Analysis of Significant Differences Between LDs and MFGs in Breast Tissue

**Figure 5:**
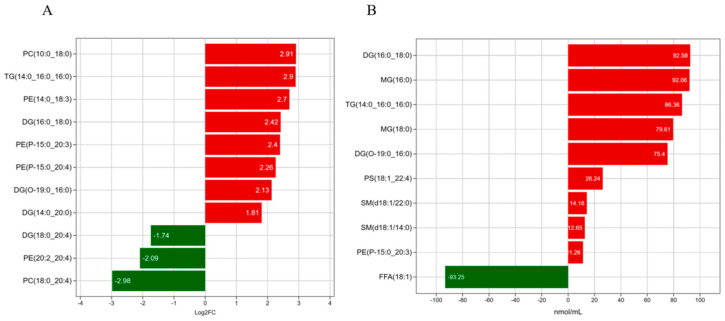
Lipid bar chart for LDs and MFGs. (**A**) Lipid difference magnitude; (**B**) changes in lipid content. Red indicates significant upregulation and green indicates significant downregulation.

The differences in TGs, DG, PC and PE between LDs and MFGs were determined, and the 10 lipids with the highest contents and number of types are shown in Figure 5A. It can be seen the largest difference in MFGs, compared with LDs, was in PC; the biggest difference was observed for phosphatidylcholine_PC (10:0_18:0), which was upregulated 2.91-fold. Phosphatidylcholine_PC (18:0_24:0) was downregulated 2.98-fold, while the content of diglycerol_DG (16:0_18:0) increased by 92.58 nmol/mL (Figure 5B), and that of oleic acid decreased by 93.25 nmol/mL. The comparison results show that there were evident differences in both the contents and number of types for four lipids, namely diglycerol (DG) (16:0:18:0), diglycerol (DG) (O-19:0:16:0), triglyceride (TG) (14:0:0:0_16:0) and phosphatidylethanolamine (PE) (P-15:0:20:3). All of these lipids were significantly upregulated, indicating that these four lipids significantly change in the transformation from mammary LDs to MFGs.

**Figure 6:**
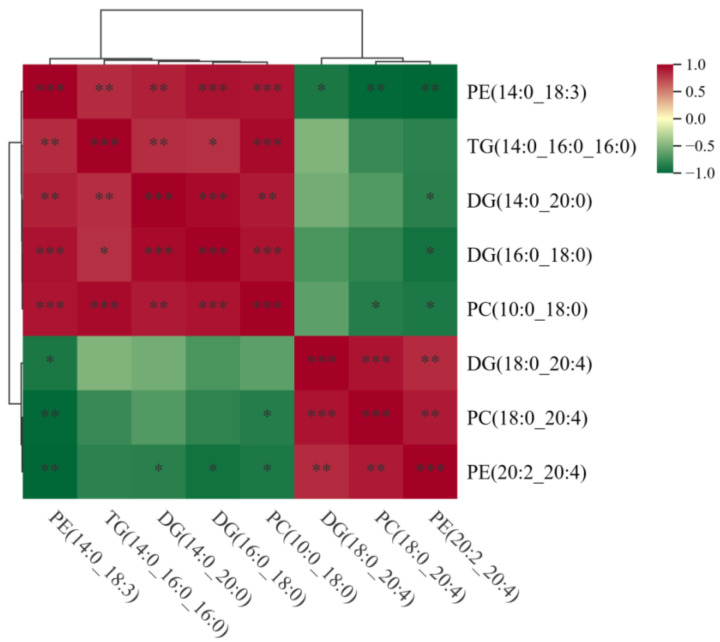
Spearman correlations of the lipids between LDs and MFGs. * *p* < 0.05, ** *p* < 0.01, *** *p* < 0.001.

To investigate the relationships among these differential lipids, Spearman’s correlations analysis was performed, as shown in Figure 6. The results indicate that PC (10:0_18:0) and PE (14:0_18:3) were significantly positively correlated with TG (14:0_16:0_16:0), DG (14:0_20:0), and DG (16:0_18:0). DG (18:0_20:4) exhibited a significant positive correlation with PC (18:0_20:4) and PE (20:2_20:4), while PE (20:2_20:4) exhibited a negative correlation with DG (14:0_20:0) and DG (16:0_18:0). These results indicate that there may be mutual conversions among PC, PE, DG and TG.

### 3.6. KEGG Pathway Analysis of Significantly Different Lipids

**Figure 7:**
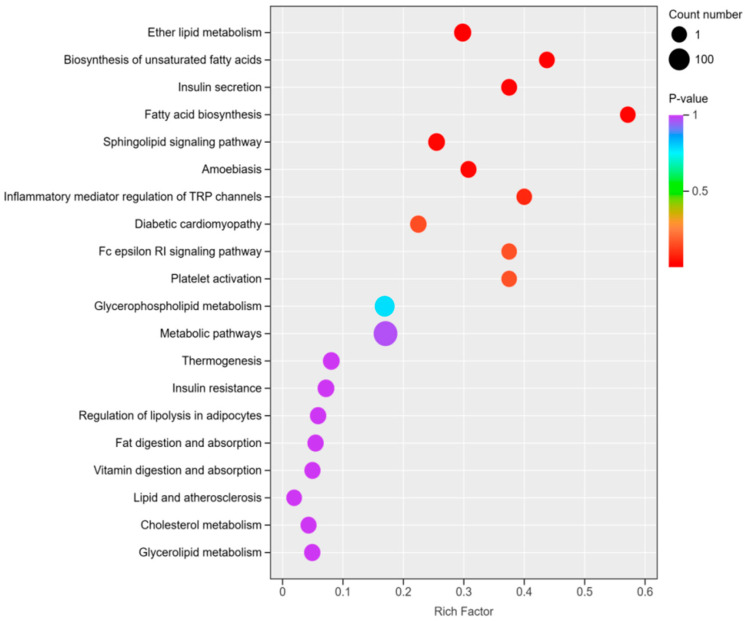
KEGG enrichment map of differential lipids between LDs and MFGs. The color of the point indicates the *p*-value, with red indicating more significant enrichment. The size of the dots represents the number of differentially enriched lipids.

In order to determine the pathway and mechanisms of the changes in significantly differentiated lipids in LDs and MFGs, KEGG pathway analysis was performed on these lipids; the results are shown in Figure 7. The enrichment factors for unsaturated fatty acid biosynthesis and fatty acid biosynthesis are the highest. A total of 152 differential lipids were enriched in metabolic pathways, and 72 differential lipids were enriched in glycerophospholipid metabolism. Ether lipid metabolism was enriched with 31 differential lipids, including DG (O-19:0:16:0) and PE (P-15:0:20:3), which are also present in the glycerol ester metabolism pathway, as are TG (14:0:0:0_16:0) and DG (16:18:0). DG (16:0_18:0), PC (10:0_18:0), DG (14:0_20:0), PE (14:0_18:3), PC (18:0_20:4), PE (20:4) and DG (18:0_20:4) are present in glycerol phosphate lipid metabolism.

## 4. Discussion

Milk contains a large amount of proteins and lipids [24]. Among these, milk fat globules (MFGs) have multiple beneficial roles and are indispensable nutrients for the growth and development of young animals [25]. Packaging fat into lipid droplets (LDs) is an evolutionary adaptation for energy storage [26], a mechanism that also offers potential strategies for preventing and treating metabolic diseases [27]. LD synthesis begins in the endoplasmic reticulum, where nascent droplets are enclosed by a phospholipid bilayer [28]. Upon secretion from the mammary gland, these LDs form milk fat globules, which are wrapped in a unique lipid–protein structure known as the MFG membrane [29,30]. However, the changes in lipid components within mammary cells during the synthesis of MFGs from LDs are unclear. Therefore, this study was designed to reveal the differences in lipid composition between LDs and MFGs from goat mammary tissue, aiming to provide new approaches for nutrition (e.g., infant formula design) and health intervention.

Lipidomics analysis revealed a total of 1167 lipid molecules in LDs and 675 lipid molecules in milk fat globules. Specifically, 259 TG species, 68 DG species, 76 PE species, and 75 PC species were identified in LDs, while 236 TG species, 28 DG species, 59 PE species, and 55 PC species were identified in MFGs. These findings are consistent with previous research [5,31], which identified 416 TG species, 15 DG species, 80 PE species, and 36 PC species in goat milk. Similarly, studies have identified 300 TG, 17 DG, 21 PC, and 14 PE species in cow milk [32]. The relative proportions of TG, PC, and PE were higher in MFGs than in LDs, whereas DG was less abundant and exhibited less variety in MFGs. These results indicate a significant remodeling of the lipid composition during the transformation of LDs into MFGs. During secretion, LDs acquire components of the mammary epithelial cell membrane, forming the distinctive three-layer membrane structure of an MFG [15]. This membrane acquisition process contributes to the observed differences in lipid composition.

Lipid droplets originate from the endoplasmic reticulum, and micro-lipid droplets carry the phospholipids and specific proteins of the ER membrane. They subsequently fuse with larger cytoplasmic lipid droplets (with diameters ranging from 1 to 15 µm), becoming the direct precursors of milk fat globules [33]. During this transformation process, the lipid components in LDs change significantly. Among GPs, PC and PE were the most abundant, with 75 types of PC in LDs, which reduced to 55 in MFGs, with its content decreasing from 4.4% to 0.9%. Moreover, the number of types of PE reduced from 76 in LDs to 59 in MFGs, and its content decreased from 0.7% to 0.3%. This result indicates that, during the transformation from LDs to MFGs, PC and PE may be hydrolyzed into other lipids. One study has also shown that PC could be hydrolyzed by diacylglycerol choline esterase [34]. Among the glycerolipids, TG is the most abundant subclass. PC is the most prevalent phospholipid in animal fat cells [15]. Consistent with these results [35], in this study, TG, DG, and PC were the three most abundant lipid classes in both LDs and MFGs. TG accounted for 4983.64 nmol/mL of the total lipid content in LDs (52.8%), increasing to 12,270.10 nmol/mL in MFGs (56.6%). The content of DG, the second most abundant glycerolipid subclass, increased from 2679.11 nmol/mL in LDd (28.4%) to 7963.28 nmol/mL in MFGs (36.7%). In contrast, the PC content decreased from 416.25 nmol/mL in LDs (4.4%) to 205.75 nmol/mL in MFGs (0.9%). These findings highlight a clear increase in TG and DG levels and a reduction in phospholipid content during LD secretion into milk. This transformation may involve processes such as LD fusion, engulfment, or lipid transfer within the mammary epithelial cells.

Pathway enrichment analysis of the differentially expressed lipids identified several key metabolic pathways, including glycerophospholipid metabolism (72 lipids), ether lipid metabolism (31 lipids), and glycerolipid metabolism (17 lipids). In the glycerophospholipid pathway, PC and PE are hydrolyzed to DG, as observed in previous studies, which demonstrated that phosphatidylcholine is converted to DG by diacylglycerol cholinephosphotransferase [36]. In the ether lipid metabolism pathway, DG (O-19:0_16:0) is enzymatically converted into ether-linked PE (PE-O), which is further transformed into PE (P-15:0_20:3). In the glycerolipid metabolism pathway, monoacylglycerols are converted into DG and TG, and TG can also be hydrolyzed to DG. PC can be hydrolyzed to DG via PLD3/4; DG is then further converted to phosphatidic acid, which is subsequently converted back to DG through PLPP or lipin proteins [37]. This pathway highlights the interconnected nature of lipid metabolism during LD secretion and MFG formation.

Considering all results of analysis of these pathways and their correlations [38], it is suggested that there may be reciprocal crosstalk and interconversion between phospholipids (e.g., PC and PE) and glycerides (e.g., DG and TG) during the secretion of lipid droplets (LDs) and their transformation into milk fat globules (MFGs). Further analysis of the 10 most significantly altered lipids revealed four key upregulated molecules with substantial changes: DG (16:0_18:0), DG (O-19:0_16:0), TG (14:0_16:0_16:0), and PE (P-15:0_20:3). These lipids appear to play critical roles in the transition of LDs into MFGs and may serve as key biomarkers for this process. In production, the milk fat content in ruminant animals significantly decreases during the lactation period by approximately 30%, with the milk production remaining unchanged. Traditionally, it was believed that the decrease in milk fat yield was caused by the dual inhibition of rumen bihydroxylation products (particularly trans-10 and cis-12 CLA) and the activity/gene expression of mammary fatty acid synthase. However, the alleviating effect on this disease was limited [39]. Our research identified many potential molecules through analyzing the differences between lipid droplets and milk fat globules. In future, these molecules can be verified through further experiments to reveal the mechanism of low-fat syndrome initiation and explore methods to improve this condition.

## 5. Conclusions

This study identified TG, DG, PC, and PE as the four most abundant lipid subclasses. Among these, specific lipid species—DG (16:0_18:0), DG (O-19:0_16:0), TG (14:0_16:0_16:0), and PE (P-15:0_20:3)—were significantly upregulated and exhibited distinct profiles, suggesting their potential role as characteristic lipid markers during the transformation of LDs into MFGs. Key lipid types, including specific diglycerides, triglycerides, and phosphatidylethanolamine, were thus implicated as being functionally important in the LD-to-MFG conversion process. These findings provide novel insights into the lipidomic differences between LDs and MFGs, and may offer a theoretical basis for understanding the molecular mechanisms underlying low-fat milk syndrome.

## Figures and Tables

**Table 1:** Lipid subclasses present in dairy goat LDs and MFGs.

Lipid Subclass	LD	MFG
Triacylglycerol (TG)	259	236
Phosphatidylethanolamine (PE)	76	59
Phosphatidylcholine (PC)	75	55
Diacylglycerol (DG)	68	28
Ceramide (Cer-NS)	65	16
Phosphatidylinositol (PI)	57	43
Phosphatidylglycerol (PG)	50	27
Free fatty acid (FFA)	44	22
Lysophosphatidylcholine (LPC)	38	14
Phosphatidylserine (PS)	31	26

## Data Availability

The datasets used and/or analyzed during the present study are available from the corresponding author upon request.
